# Dual effects of the small-conductance Ca^2+^-activated K^+^ current on human atrial electrophysiology and Ca^2+^-driven arrhythmogenesis: an in silico study

**DOI:** 10.1152/ajpheart.00362.2023

**Published:** 2023-08-25

**Authors:** Nathaniel T. Herrera, Xianwei Zhang, Haibo Ni, Mary M. Maleckar, Jordi Heijman, Dobromir Dobrev, Eleonora Grandi, Stefano Morotti

**Affiliations:** ^1^Department of Pharmacology, University of California Davis, Davis, California, United States; ^2^Department of Computational Physiology, Simula Research Laboratory, Oslo, Norway; ^3^Department of Cardiology, Faculty of Health, Medicine, and Life Sciences, Cardiovascular Research Institute Maastricht, Maastricht University, Maastricht, The Netherlands; ^4^Faculty of Medicine, West German Heart and Vascular Center, Institute of Pharmacology, University Duisburg-Essen, Essen, Germany; ^5^Department of Medicine, Montreal Heart Institute and Université de Montréal, Montreal, Quebec, Canada; ^6^Department of Integrative Physiology, Baylor College of Medicine, Houston, Texas, United States

**Keywords:** arrhythmia, atrial fibrillation, atrial myocyte electrophysiology, mathematical model, spontaneous Ca^2+^ release

## Abstract

By sensing changes in intracellular Ca^2+^, small-conductance Ca^2+^-activated K^+^ (SK) channels dynamically regulate the dynamics of the cardiac action potential (AP) on a beat-to-beat basis. Given their predominance in atria versus ventricles, SK channels are considered a promising atrial-selective pharmacological target against atrial fibrillation (AF), the most common cardiac arrhythmia. However, the precise contribution of SK current (*I*_SK_) to atrial arrhythmogenesis is poorly understood, and may potentially involve different mechanisms that depend on species, heart rates, and degree of AF-induced atrial remodeling. Both reduced and enhanced *I*_SK_ have been linked to AF. Similarly, both SK channel up- and downregulation have been reported in chronic AF (cAF) versus normal sinus rhythm (nSR) patient samples. Here, we use our multiscale modeling framework to obtain mechanistic insights into the contribution of *I*_SK_ in human atrial cardiomyocyte electrophysiology. We simulate several protocols to quantify how *I*_SK_ modulation affects the regulation of AP duration (APD), Ca^2+^ transient, refractoriness, and occurrence of alternans and delayed afterdepolarizations (DADs). Our simulations show that *I*_SK_ activation shortens the APD and atrial effective refractory period, limits Ca^2+^ cycling, and slightly increases the propensity for alternans in both nSR and cAF conditions. We also show that increasing *I*_SK_ counteracts DAD development by enhancing the repolarization force that opposes the Ca^2+^-dependent depolarization. Taken together, our results suggest that increasing *I*_SK_ in human atrial cardiomyocytes could promote reentry while protecting against triggered activity. Depending on the leading arrhythmogenic mechanism, *I*_SK_ inhibition may thus be a beneficial or detrimental anti-AF strategy.

**NEW & NOTEWORTHY** Using our established framework for human atrial myocyte simulations, we investigated the role of the small-conductance Ca^2+^-activated K^+^ current (*I*_SK_) in the regulation of cell function and the development of Ca^2+^-driven arrhythmias. We found that *I*_SK_ inhibition, a promising atrial-selective pharmacological strategy against atrial fibrillation, counteracts the reentry-promoting abbreviation of atrial refractoriness, but renders human atrial myocytes more vulnerable to delayed afterdepolarizations, thus potentially increasing the propensity for ectopic (triggered) activity.

## Graphical abstract

**Figure:**
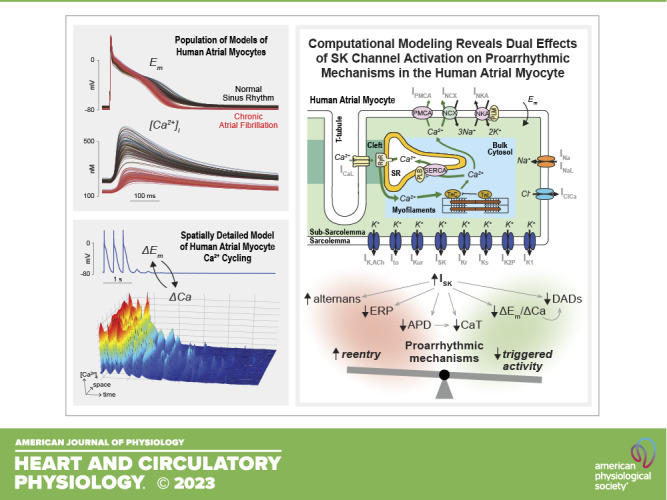

## INTRODUCTION

Atrial fibrillation (AF) is the most common clinical arrhythmia that significantly affects cardiovascular health worldwide (1, 2). It is characterized by rapid and irregular electrical activity and nonuniform electrical conduction leading to compromised atrial function. This impairment is linked to an enhanced risk of stroke and contributes to cardiovascular morbidity and mortality. The mechanisms underlying AF are complex and involve both ectopic (triggered) activity from atrial foci (triggers) and a vulnerable substrate favoring impulse reentry through atrial tissue (3, 4). At the cellular level, focal ectopic/triggered activity is likely caused by early and delayed afterdepolarizations (EADs and DADs) or enhanced automaticity, while reentry is promoted by shortening of the action potential (AP) duration (APD), abbreviated atrial myocyte refractoriness (effective refractory period, ERP), and increased spatial and temporal heterogeneity (APD/ERP dispersion and alternans). In recent years, a large body of work has provided valuable insight in the molecular and cellular underpinnings of AF pathophysiology (5). Understanding the mechanisms underlying AF can facilitate targeted interventions, but at present pharmacological options demonstrate poor efficacy and safety concerns, and ablation is still the most effective clinical treatment (5). It is generally assumed that pharmacological treatment may be particularly suitable during the early stage of AF (i.e., paroxysmal AF), before significant electrical, structural, Ca^2+^ handling, and contractile remodeling that can lead to long-standing persistent (chronic) AF (cAF) (6, 7). To avoid malignant adverse effects on ventricular electrophysiology, current research aims at exploiting atrioventricular electrophysiological differences to develop AF-selective strategies (8–11). For example, pharmacological approaches based on AF-selective blockade of the fast Na^+^ current (*I*_Na_) have been proposed (12, 13), as well as those targeting K^+^ channels primarily expressed in the atria (e.g., acetylcholine-sensitive and ultrarapid K^+^ currents, *I*_K,ACh_ and *I*_Kur_) (9, 11).

Given their predominant expression in atria versus ventricles, small-conductance Ca^2+^-activated K^+^ (SK) channels are emerging as a promising atrial-selective pharmacological target against AF (14–16). The family of SK channels consists of three members (17): SK1, SK2, and SK3, encoded by different genes (KCNN1, KCNN2, and KCNN3, respectively) and characterized by different sensitivity to the specific and potent channel pore blocker apamin (18). SK channels have been widely studied in the past two decades, since their identification in the heart and the assessment of their functional role in AP repolarization (17, 19). These initial studies showed that SK current (*I*_SK_) shortens atrial (but not ventricular) APD under physiological conditions. *I*_SK_ is modulated by Ca^2+^ (mediated by calmodulin tethered to the channel) (20) and can serve as an efficient process linking changes in intracellular Ca^2+^ cycling and sarcoplasmic reticulum (SR) Ca^2+^ release to transmembrane potential (*E*_m_) dynamics (21). Despite the lack of a voltage sensor in the channel, inwardly rectifying *I*_SK_-voltage relationships have been observed in many studies in both native and cloned channels from various species (22–25), and attributed to a voltage-dependent modulation of SK channel pore conductance by intracellular divalent (Ca^2+^ and Mg^2+^) cations (26–29). Recent data revealed that SK channel trafficking also increases in a Ca^2+^-dependent manner (30, 31). These properties make SK channels of particular interest during fast pacing or tachyarrhythmias (like AF), when Ca^2+^ accumulation is likely to enhance their activation. An increase in *I*_SK_ is expected to hasten repolarization, thus limiting APD and Ca^2+^ accumulation (a protective effect) but also to shorten the ERP, which may facilitate the formation of reentry circuits (proarrhythmic). Moreover, an altered dynamic APD regulation at fast rates may influence the induction of alternans (32), possibly promoting reentrant arrhythmias.

Indeed, both SK channel hyperactivity (24, 31, 33–40) and suppression (22, 25, 41) have been implicated in AF, likely through distinct mechanisms, and depending on species, heart rates, and disease etiology. Here, we aim to investigate the impact of modulating *I*_SK_ on the electrophysiology of human atrial myocytes and their vulnerability to Ca^2+^-driven arrhythmias using mathematical modeling and simulation, which have proven useful tools for gaining mechanistic insight into AF mechanisms and developing novel antiarrhythmic pharmacological approaches (4, 42). Our findings contribute to a better quantitative understanding of the role of *I*_SK_ in various arrhythmia-provoking contexts, shed light on the underlying arrhythmia mechanisms, and provide insights into the potential proarrhythmic or protective effects of SK channel modulation in AF.

## METHODS

### Human Atrial Cardiomyocyte Simulations

To investigate the impact of SK channel modulation on atrial electrophysiology and arrhythmogenesis, we updated our established model of the human atrial myocyte in nSR and cAF conditions (43–45). Note that this model does not account for the sex-specific formulation of atrial myocyte electrophysiology and Ca^2+^ handling, as model parameters have been previously fitted and validated against experimental data that included both male and female patients. We included in this cellular framework the *I*_SK_ formulation that we have recently developed based on recordings in human right-atrial cardiomyocytes from patients with nSR and cAF (31). To simulate *I*_Na_, we used the formulation developed by Courtemanche et al. (46) and adjusted its maximal conductance to maintain the physiological AP upstroke velocity. The formulation of the late Na^+^ current (*I*_NaL_) was modified by reducing the time constant of inactivation to reproduce experimental data at physiological temperature (47), as previously described (48). We also introduced a 21-mV rightward shift in the voltage dependence of the inactivation of *I*_NaL_ to match the half-activation potential of *I*_Na_ (46), and then adjusted *I*_NaL_ maximal conductance to reproduce the same current density during the AP. Lastly, we modified the cAF parameterization used in Ni et al. (45) by increasing the sensitivity of ryanodine receptor (RyR) for SR Ca^2+^ levels (i.e., 50% reduction in the EC_50_ for [Ca^2+^]_SR_-dependent activation of SR Ca^2+^ release) compared with the baseline nSR condition. Our default cAF model does not account for any AF-associated SK channel remodeling. However, we performed a separate set of simulations in which both SK channel conductance and Ca^2+^ sensitivity were altered as seen in AF (31, 49).

To gain a deeper understanding of the processes regulating Ca^2+^-driven arrhythmias, we also performed a set of simulations with our 3-D model of the human atrial myocyte integrating membrane electrophysiology, spatially detailed Ca^2+^ handling, and variable tubular network (50, 51). We constructed 10 different cardiomyocyte models generating different ultrastructures characterized by comparable overall low tubular densities, as previously described (50).

### Electrophysiological Protocols

The effect of modulation of the maximal conductance of SK channels (G_SK_) on AP and Ca^2+^ transient (CaT) properties at different pacing rates (i.e., between 0.5 and 4 Hz, with 0.5-Hz increments) was estimated at steady state for both nSR and cAF. To investigate how G_SK_ changes affect atrial refractoriness, we determined the ERP simulating an S1–S2 premature stimulation protocol. First, for each condition studied, we identified the diastolic threshold of excitation (DTE). The S1 stimulus (5 ms in duration, 2-fold the DTE in amplitude) was applied at a basic cycle length (BCL) until steady state. To determine the ERP, we then applied the premature S2 stimulus (same duration and amplitude as S1) at progressively smaller S1–S2 intervals by decrements of 5 ms. As previously described (52), the longest S1–S2 interval that failed to elicit an AP was taken as the local ERP (i.e., maximum upstroke velocity of ≥5 V/s and AP amplitude of ≥50% of that of the preceding AP elicited by S1).

We studied the role of G_SK_ modulation in Ca^2+^-driven atrial arrhythmias by investigating the determinants of alternans and DADs. The occurrence of alternans was assessed at steady state. By progressively increasing the stimulation frequency (1-ms decrements in BCL), we identified the maximum pacing thresholds required for the development of voltage (i.e., causing a ≥5 ms difference in APD estimated at *E*_m_ = −60 mV in subsequent beats) and Ca^2+^- (i.e., causing a ≥10 nM difference in CaT amplitude, CaT_amp_, in subsequent beats) driven alternans. The occurrence of DADs was assessed by stimulating the models at a fast rate for 10 s in the presence of 1 µM isoproterenol, and then pausing the stimulation. By progressively increasing the stimulation frequency (1-ms decrements in BCL), we identified the maximum pacing threshold required for the development of DADs (i.e., causing a voltage oscillation ≥10 mV during the pause).

We used the group of 10 3-D models to assess the effect of G_SK_ modulation on spontaneous Ca^2+^ release events (SCRs) and consequent DADs at different pacing rates (i.e., 0.5, 1, 2, 3, 4, and 5 Hz). Each simulation consisted of a quiescent period of 4 s, a pacing period of 28 s to achieve a steady state and a final quiescent period of 5 s for observation of oscillations in *E*_m_ and global [Ca^2+^]_i_. Whenever present, we quantified the amplitude of the voltage oscillation (Δ*E*_m_) by measuring the difference between the first peak and diastolic minimum during the final quiescent period. Then, we identified the corresponding SCR as the nearest [Ca^2+^]_i_ peak preceding the voltage oscillation and determined its amplitude (ΔCa) by calculating the difference between the peak and diastolic minimum during the final quiescent period. We used cutoff values of 300 nM and 10 mV for oscillations in [Ca^2+^]_i_ and *E*_m_, respectively.

### Populations of Human Atrial Cardiomyocyte Models

Following an established approach (53), we built two populations of 1,000 atrial myocytes in nSR and cAF conditions by randomly perturbing the parameters of the Grandi et al. model listed in Table A1 with log-normal scaling factors (σ = 0.1). Properties of AP and CaT, ERP, and maximum pacing thresholds for alternans and DADs were determined for each model variant in the two populations, as aforementioned. To quantify the impact of parameter perturbations on the features analyzed, we performed multivariable linear and logistic regression analyses (53–55). Population size and parameter perturbation variance were chosen to ensure convergence of the results of these sensitivity analyses, as previously discussed (45, 56, 57).

### Numerical Methods and Code

Simulations of the updated Grandi et al. model were performed with a standard desktop using MATLAB (MathWorks, Natick, MA), v. R2022a. Simulations of the Zhang et al. 3-D model (implemented in C++ and parallelized using OpenMP 5.1) were performed with a computing cluster with Intel Xeon CPU E5-2690 v4 at 2.60 GHz 28 CPUs (56 threads) + 132 GB. Data analysis was performed with MATLAB using a standard desktop. Model codes are freely available on the authors’ website (see DATA AVAILABILITY).

## RESULTS

**Figure 1.**
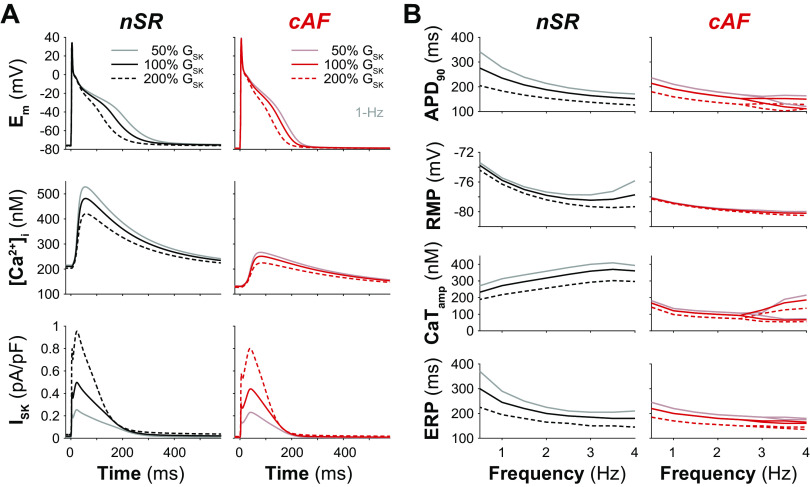
Increasing SK channel conductance shortens human atrial myocyte action potential duration (APD) and effective refractory period (ERP) in normal sinus rhythm (nSR) and chronic atrial fibrillation (cAF). *A*: time course of membrane potential (*E*_m_), cytosolic Ca^2+^ concentration ([Ca^2+^]_i_), and SK current (*I*_SK_) in nSR and cAF myocytes during 1-Hz pacing for different values of SK channel maximal conductance (G_SK_). *B*: effects of G_SK_ modulation on frequency dependence of APD at 90% repolarization (APD_90_), resting membrane potential (RMP), Ca^2+^ transient amplitude (CaT_amp_), and ERP in nSR and cAF myocytes. Note that, when the cAF model develops alternans (i.e., at pacing rates of ≥3 Hz), values obtained at two subsequent alternating beats are reported.

Our study used the Grandi et al. model of the human atrial cardiomyocyte in nSR and cAF (43), with subsequent modifications made as described in previous work (44, 45) and also detailed in methods. We first quantified the impact of G_SK_ modulation (set to 50%, 100%, and 200% of the nominal value) on steady-state AP and CaT properties at varying stimulation frequencies (Fig. 1). We chose these G_SK_ values based on the range of variation experimentally observed, adopting, as previously done (45), a 1:2 ratio that approximates the reported AF-dependent decrease (58) and average increase in *I*_SK_ (24, 31, 59). In the absence of G_SK_ perturbation (i.e., 100% G_SK_), the cAF model displays shorter AP and ERP, and a more negative (polarized) resting *E*_m_ (RMP) compared with nSR. Despite the depressed cytosolic CaT, *I*_SK_ peak in cAF is comparable to that in nSR when the conductance does not undergo arrhythmia-driven remodeling (Fig. 1*A*). This is due to the fact that SK channel function is controlled by cleft and subsarcolemmal Ca^2+^ levels (i.e., not cytosolic), which rise both well above the *K*_d_ used to model Ca^2+^ activation of *I*_SK_ (*K*_d,SK_ = 350 nM) during the AP (not shown). The described differences between nSR and cAF AP and CaT features are maintained in the presence of G_SK_ perturbations. Our simulations show that increasing G_SK_ shortens the atrial AP and ERP, hyperpolarizes RMP, and reduces CaT_amp_ in both nSR and cAF myocytes (Fig. 1*B*). Simulations of the cAF model revealed the occurrence of alternans at fast pacing rates, independently of the G_SK_ value in use (Fig. 1*B*).

**Figure 2.**
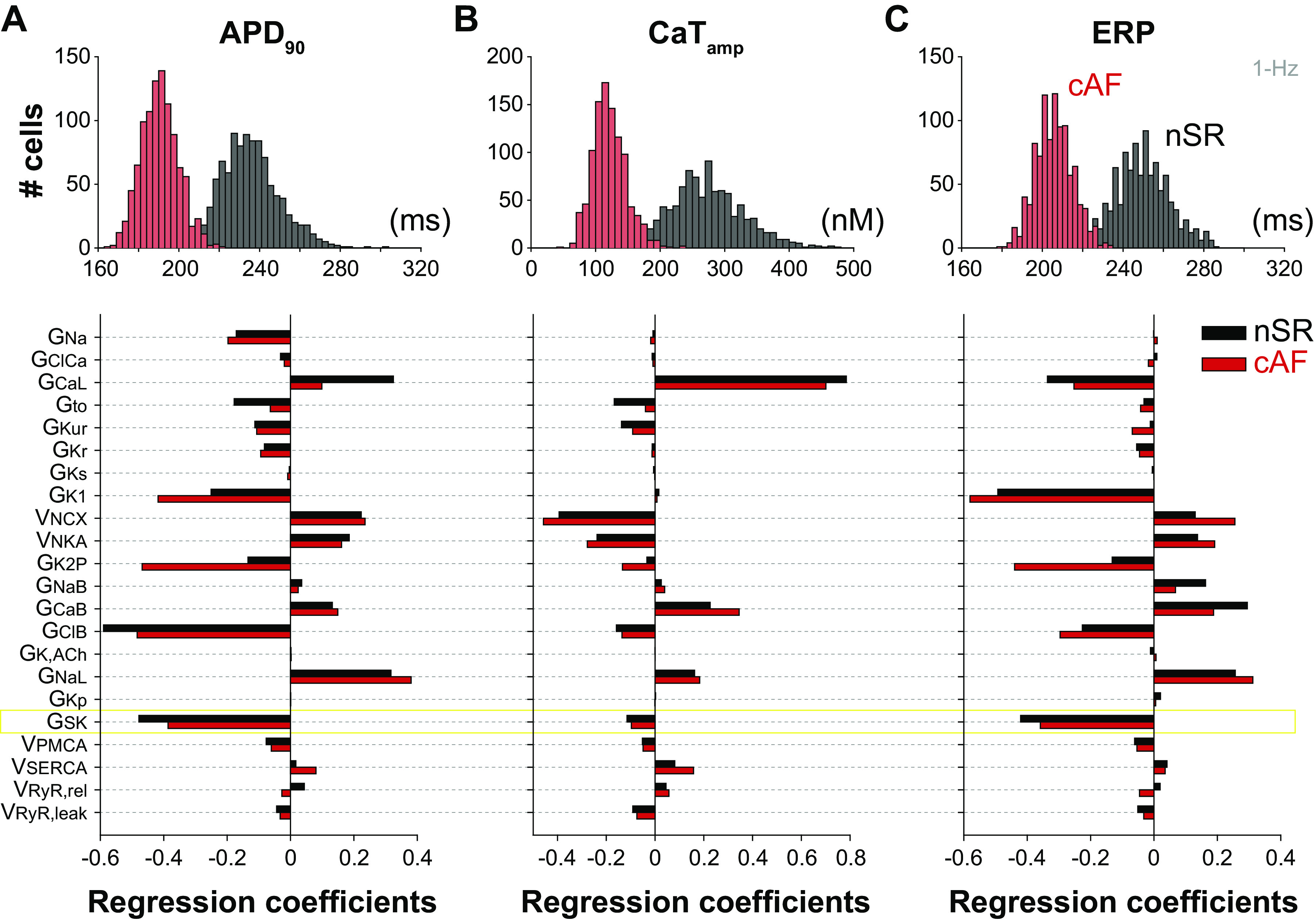
Sensitivity analysis reveals that SK channel conductance has a prominent role in modulating atrial myocyte action potential duration (APD) and effective refractory period (ERP). *Top* panels report histograms showing the distribution of APD_90_ (*A*), CaT_amp_ (*B*), and ERP (*C*) assessed at 1-Hz pacing in nSR and cAF populations of 1,000 models. *Bottom* panels report the results of linear regression analysis performed to quantify the sensitivity of APD_90_, CaT_amp_, and ERP (assessed at 1-Hz pacing) to changes in the listed individual parameters in the nSR and cAF models. Simulated data were obtained in nSR and cAF populations built upon baseline models with nominal SK channel maximal conductance (i.e., 100% G_SK_). Model variants exhibiting AP irregularities (1 in nSR, 4 in cAF) were excluded from linear regression analysis. APD_90_, APD at 90% repolarization; cAF, chronic atrial fibrillation; CaT_amp_, Ca^2+^ transient amplitude; nSR, normal sinus rhythm.

To quantify the impact of small G_SK_ changes on human atrial myocyte electrophysiology and assess the relative role of *I*_SK_ compared with other currents and transporters, we performed a parameter sensitivity analysis adopting an established methodology (53) based on the “population of models” approach (60). By randomly varying the values of maximal conductance and transport rate of ion channels and transporters (defined in Table A1), we generated two populations of atrial cells in nSR and cAF conditions reproducing the natural cell-to-cell variability in myocyte properties (see Fig. 2, *top*). We applied multivariable linear regression to quantify the sensitivity of APD, CaT_amp_, and ERP (assessed in each model variant at 1-Hz pacing) to changes in the perturbed model parameters (53). Our analysis revealed a predominant role of G_SK_ in modulating atrial myocyte APD and refractoriness (Fig. 2, *A* and *C*), with a modest contribution to the regulation of CaT_amp_ (Fig. 2*B*). The coefficients associated to G_SK_ are negative, which indicates that increasing this parameter causes a reduction in APD, CaT_amp_, and ERP. The large amplitude of the G_SK_ coefficients for APD and ERP indicates that these features are highly sensitive to G_SK_ in both nSR and cAF, along with other K^+^ (i.e., basal inward rectifier and two-pore domain currents, *I*_K1_ and *I*_K2P_) and background Cl^−^ currents. CaT_amp_ is less sensitive to K^+^ currents and primarily affected by changes in maximal conductance of L-type Ca^2+^ channels (G_CaL_) and transport rates of Na^+^/Ca^2+^ exchanger (v_NCX_) and Na^+^/K^+^ ATPase (v_NKA_), which are also positively correlated with APD changes, along with G_NaL_. Taken together, our results suggest that increased *I*_SK_ mainly causes APD and ERP shortening. These alterations have the potential to promote arrhythmogenesis as they increase the susceptibility to reentry, which can initiate arrhythmia within the atrial tissue and contribute to its persistence over time (4).

**Figure 3.**
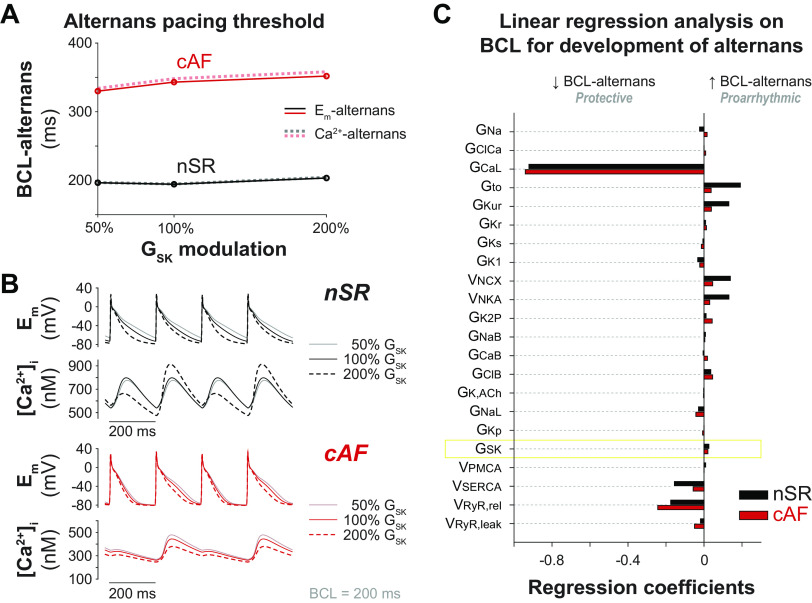
Increasing SK channel conductance modestly favors development of alternans in nSR and cAF myocytes. *A*: effect of G_SK_ modulation on the maximal basic cycle length (BCL) inducing voltage (solid lines)- and Ca^2+^ (dotted lines)-driven alternans in the baseline nSR and cAF models. *B*: time course of membrane potential and cytosolic Ca^2+^ concentration obtained at steady state in the baseline nSR and cAF models during 5-Hz pacing with variable SK channel maximal conductance. *C*: results of linear regression analysis performed to quantify the sensitivity of maximal BCL for voltage-driven alternans development (BCL alternans) to changes in the listed model parameters in nSR and cAF. Simulated data were obtained in populations of 1,000 nSR and cAF models built upon baseline models with nominal SK channel maximal conductance (i.e., 100% G_SK_). Model variants exhibiting alternans at 1-Hz pacing rate (1 in nSR, 4 in cAF) and model variants that never exhibited alternans (14 in nSR) were excluded from the linear regression analysis, which was performed analyzing the remaining 985 nSR and 996 cAF models. cAF, chronic atrial fibrillation; nSR, normal sinus rhythm.

When pacing the cAF model at fast rates, our simulations revealed the development of alternans (Fig. 1*B*), another well-known contributor to reentrant arrhythmias (4). To identify the contribution of *I*_SK_ to this mechanism, we first assessed the maximal BCL at which alternans occur in the baseline nSR and cAF models (Fig. 3, *A* and *B*). We separately determined BCL thresholds for the development of beat-to-beat variations in APD (for simplicity called voltage-driven alternans) and CaT_amp_ (Ca^2+^-driven alternans). In agreement with the results shown in Fig. 1, BCL thresholds are longer in cAF versus nSR (343 and 348 ms vs. 194 and 195 ms at 100% G_SK_ for voltage- and Ca^2+^-driven alternans, respectively), indicating that propensity for alternans is enhanced in disease. Although G_SK_ changes minimally alter BCL thresholds in nSR (197/197 and 203/204 ms at 50% and 200% G_SK_, respectively, for voltage/Ca^2+^-driven alternans), increasing G_SK_ moderately elevates the BCL thresholds in cAF (330/334 and 352/358 ms at 50% and 200% G_SK_, respectively, for voltage/Ca^2+^-driven alternans). We then determined the BCL thresholds for voltage-driven alternans in each model variant of our nSR and cAF populations and performed multivariable linear regression (53) to quantify how changes in model parameters alter this threshold (Fig. 3*C*). The positive regression coefficients determined for G_SK_ indicate that increasing this parameter prolongs the BCL for alternans in both nSR and cAF, increasing the vulnerability to reentry. However, the small amplitude of these coefficients (with respect to those of other coefficients) indicates that the contribution of *I*_SK_ to the development of alternans is limited compared with that of other channels or transporters. Our results show that the parameters that are more influential for the pacing threshold for alternans in both nSR and cAF are G_CaL_ and the maximal rate of SR Ca^2+^ release through RyRs (v_RyR,rel_). Increasing these parameters would reduce the pacing threshold, thereby protecting against alternans.

**Figure 4.**
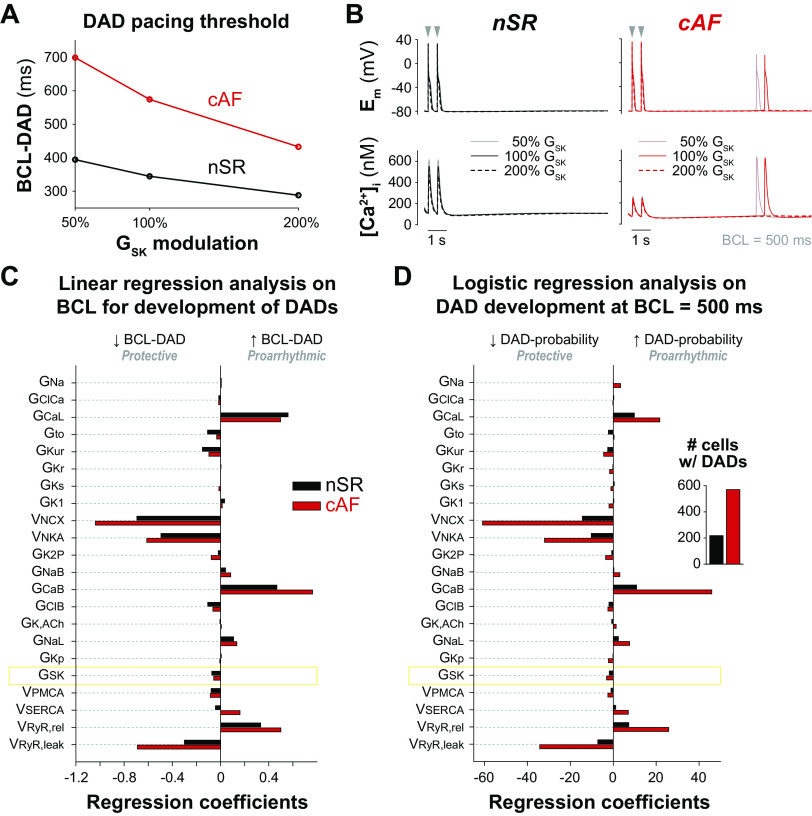
Increasing SK channel conductance protects against delayed afterdepolarization (DAD) development in nSR and cAF myocytes. *A*: effect of G_SK_ modulation on the maximal basic cycle length (BCL) inducing DADs in the baseline nSR and cAF models. *B*: time course of membrane potential and cytosolic Ca^2+^ concentration elicited in the baseline nSR and cAF models after pausing the electrical stimulation after 10 s of 2-Hz pacing in the presence of Isoproterenol (1 µM) with variable G_SK_. Only the last two paced beats are reported in the figure. *C*: results of linear regression analysis quantifying the sensitivity of maximal BCL for DAD development (BCL-DAD) to changes in the listed model parameters in nSR and cAF. *D*: results of logistic regression analysis quantifying the impact of the listed model parameters on the probability of DAD development in nSR and cAF at 2-Hz pacing (i.e., BCL = 500 ms). Intercept terms b_0_ (determining the probability of DAD development in the absence of parameter perturbations) are −19.1 for nSR and +12.7 for cAF. *Inset*: number of model variants showing DADs during 2-Hz pacing within the nSR and cAF populations. Simulated data analyzed in *C* and *D* were obtained in populations of 1,000 nSR and cAF models built upon baseline models with nominal SK channel maximal conductance (i.e., 100% G_SK_). Model variants exhibiting DADs at 1-Hz pacing rate (129 in nSR, 422 in cAF) and model variants that never exhibited DADs (24 in nSR, 62 in cAF) were excluded from the linear regression analysis shown in *C*, which was performed analyzing the remaining 847 nSR and 516 cAF models. cAF, chronic atrial fibrillation; nSR, normal sinus rhythm.

We next assessed the involvement of *I*_SK_ in the development of DADs, a Ca^2+^-driven phenomenon that can cause triggered activity, thereby contributing to AF initiation (4). We first assessed the impact of G_SK_ modulation on the maximal BCL at which DADs occur in our baseline nSR and cAF models (Fig. 4, *A* and *B*). BCL thresholds are higher in cAF versus nSR (574 vs. 345 ms at G_SK_ 100%), confirming the disease-associated increase in DAD propensity. Furthermore, increasing G_SK_ reduces the BCL thresholds in both nSR (394 and 288 ms at 50% and 200% G_SK_, respectively) and cAF (699 and 432 ms at 50% and 200% G_SK_, respectively), thereby protecting against arrhythmias. As described for alternans, we determined the BCL thresholds for DADs in each model in our populations and performed linear regression (53) to quantify how changes in model parameters alter the propensity for DADs (Fig. 4*C*). The negative regression coefficients determined for G_SK_ indicate the protective effect of *I*_SK_ activation against DADs in both nSR and cAF (i.e., increasing G_SK_ shortens the BCL for DADs). Our analysis also highlights that the sensitivity to changes in G_SK_ is limited if compared with changes in other parameters that are directly involved in the regulation of Ca^2+^ cycling, such as G_CaL_, v_NCX_, v_NKA_, and v_RyR,rel_. We next separated our nSR and cAF populations in two subgroups of model variants based on the development of DADs at 2-Hz pacing (i.e., models with a BCL threshold ≥500 ms). Using this cutoff, we identified 218 and 570 model variants exhibiting DADs within the nSR and cAF populations, respectively (Fig. 4*D*, *inset*). As previously described (55), we performed multivariable logistic regression to quantify how changes in model parameters affect the probability of DAD development in nSR and cAF. Our results show a negative correlation between G_SK_ modulation and DAD probability (Fig. 4*D*), confirming that *I*_SK_ activation is protective against DADs. This analysis also shows that modulation of G_SK_ has a smaller effect on DAD probability compared with changes in G_CaL_, v_NCX_, v_NKA_, and v_RyR,rel_, corroborating the findings obtained with linear regression (Fig. 4*C*).

**Figure 5.**
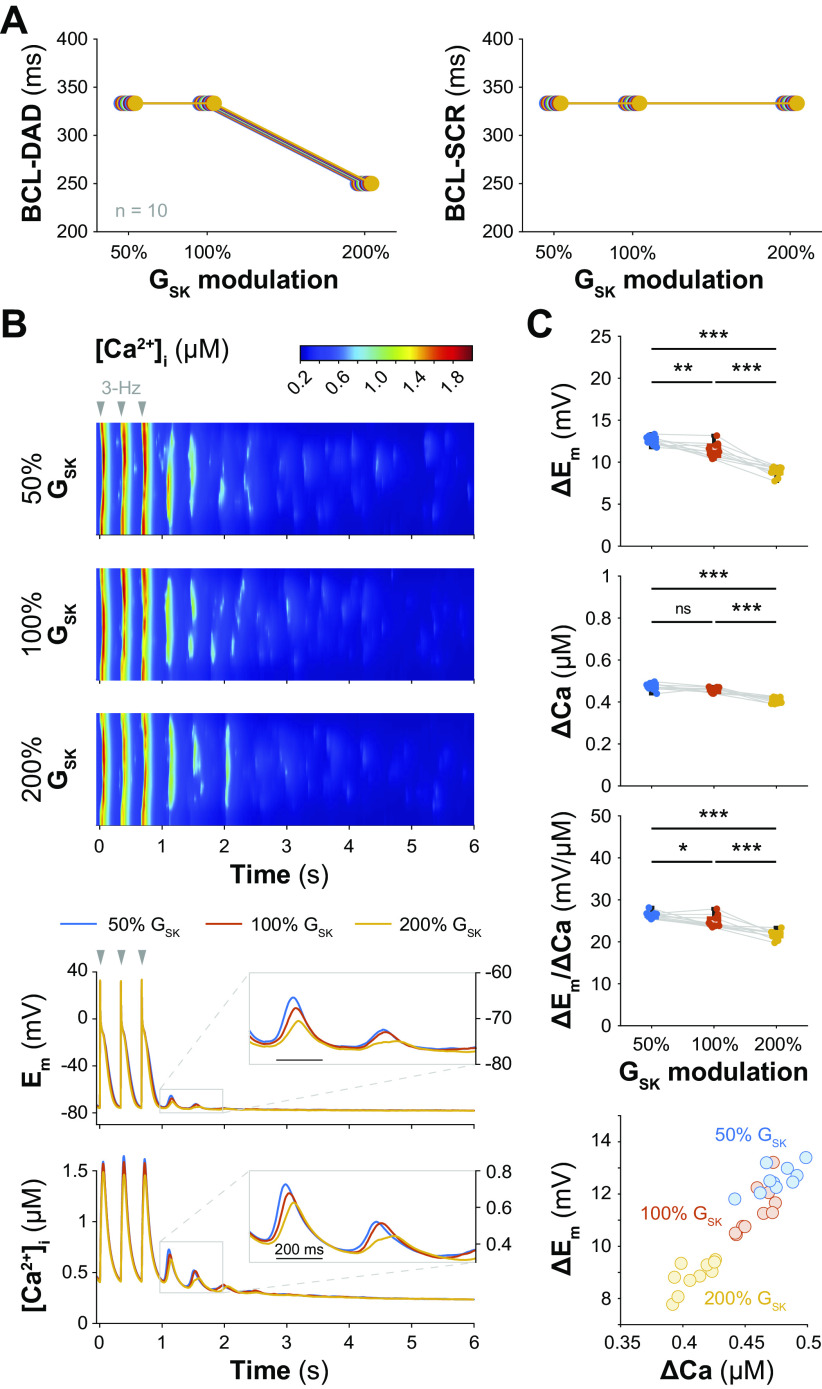
Increasing SK channel conductance protects against DADs by enhancing the repolarizing force that counteracts the Ca^2+^-dependent depolarization. *A*: effect of changes in G_SK_ on the maximal basic cycle length (BCL) inducing DADs and spontaneous Ca^2+^ release events (SCRs) in a human atrial myocyte model with 3-D Ca^2+^ diffusion. Thresholds for DADs and SCRs were set to 10 mV and 300 nM, respectively. The simulations were repeated for 10 randomly generated tubular structures. *B*, *top*: representative transverse line scans of local cytosolic Ca^2+^ concentration obtained pausing the electrical stimulation after a train of impulses at 3-Hz pacing for different G_SK_ values. The last three paced beats are reported in the figure. *B, bottom*: time course of corresponding global cytosolic Ca^2+^ concentration and membrane potential. *C*: summary data resulting from simulating the protocol described in *B* in the 10 different tubular structures. *Top* panels show amplitude of *E*_m_ and [Ca^2+^]_i_ oscillations (Δ*E*_m_ and ΔCa), and the Δ*E*_m_/ΔCa ratio in function of different G_SK_ values. Statistical analysis was performed by one-way ANOVA with Bonferroni correction (***P < 0.001; **P < 0.01; *P < 0.05; ns: not significant). Δ*E*_m_ and ΔCa are also compared against each other in the scatter plot reported in the *bottom* panel. ANOVA, analysis of variance; DAD, delayed afterdepolarization.

To get mechanistic insight into the subcellular processes involved in the development of triggered activity, we simulated our recently developed 3-D model of the human atrial myocyte with spatially detailed Ca^2+^ cycling (50, 51). We constructed 10 different cardiomyocyte models characterized by similar low density but different organization of the transverse tubular network, and assessed the relationship between the occurrence of SCRs and DADs and the pacing rate. In agreement with our previous results, we found that an increase in G_SK_ is protective against DADs (i.e., lower BCL threshold, see Fig. 5*A*, *left*). Interestingly, the BCL threshold for SCR is not affected by G_SK_ modulation (Fig. 5*A*, *right*), suggesting altered coupling between *E*_m_ and [Ca^2+^]_i_ with increased G_SK_. To confirm this hypothesis, we analyzed both supra- and subthreshold *E*_m_ and [Ca^2+^]_i_ oscillations obtained after stimulating the cells at a pacing frequency of 3 Hz (Fig. 5, *B* and *C*). We found that increasing G_SK_ significantly reduces the amplitude of *E*_m_ oscillations (Δ*E*_m_). The amplitude of the [Ca^2+^]_i_ oscillations (ΔCa) is not different between 50% and 100% G_SK_, but is significantly reduced with 200% G_SK_. To assess changes in *E*_m_-Ca^2+^ coupling, we calculated the Δ*E*_m_/ΔCa ratio and identified a significant reduction with an increase in G_SK_. Notably, we obtained similar results when investigating different pacing rates between 2 and 5 Hz (Supplemental Fig. S1; note: Supplemental data may be found at https://doi.org/10.6084/m9.figshare.23982561.v1). Taken together, these results indicate that the protective effect of G_SK_ activation against the development of DADs is due to a strengthening of the repolarizing force that counteracts the Ca^2+^-dependent depolarization.

## DISCUSSION

**Figure 6.**
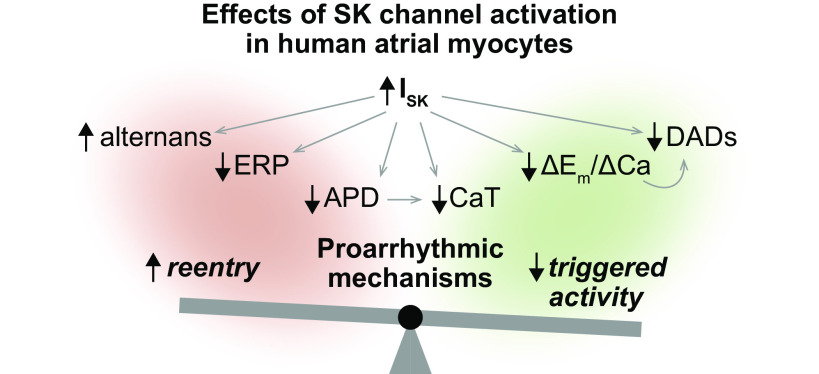
Dual effects of SK channel activation in human atrial myocytes. Our simulations indicate that increased SK current promotes *1)* APD and ERP shortening, *2)* slight increase in propensity for alternans, *3)* depressed Ca^2+^ cycling, *4)* decrease in Δ*E*_m_/ΔCa ratio (i.e., enhanced repolarizing force), and *5)* reduced propensity for DADs. Although effects *1* and *2* promote the development of an arrhythmogenic reentrant substrate, effects *3*, *4*, and *5* counteract triggered activity. APD, action potential duration; DAD, delayed afterdepolarization; ERP, effective refractory period.

We performed a computational analysis aimed at unraveling the role of SK channels in human atrial myocyte electrophysiology and arrhythmogenesis. Using our established multiscale modeling framework, we simulated various electrophysiology protocols to determine the impact of *I*_SK_ modulation on the regulation of APD, CaT, and ERP, as well as the occurrence of arrhythmogenic alternans and DADs. Our findings indicate that increased G_SK_ promotes APD and ERP shortening, while slightly increasing the propensity for alternans, thereby primarily promoting the development of an arrhythmogenic reentrant substrate. Our simulations also demonstrated that enhanced *I*_SK_ limits Ca^2+^ cycling and counteract the occurrence of DADs, thus exerting a protective effect against triggered activity. This protective effect is due to an enhanced repolarizing force (i.e., reduced Δ*E*_m_/ΔCa ratio) when G_SK_ is increased. Overall, these results (summarized in Fig. 6) highlight the potential dual effect of targeting SK channels for the treatment of AF depending on the leading arrhythmogenic mechanism. These observations align with existing literature reporting that both reduced and enhanced *I*_SK_ may predispose to AF in animal models (22, 24, 25, 33–41). As discussed below, these contrasting outcomes of *I*_SK_ modulation can be attributed to species differences, variability in proarrhythmic protocols, as well as the extent and type of AF-induced atrial remodeling.

### SK Contribution to AF Remodeling and Arrhythmogenesis

Progression of AF is associated with extensive remodeling of ion channels and Ca^2+^ handling proteins (5). A genome-wide association study suggested a possible link between human lone AF and an intronic single nucleotide polymorphism in the KCNN3 gene (61, 62), but the precise role of SK channels in atrial arrhythmogenesis is still poorly defined (14–16). Indeed, both *I*_SK_ upregulation and inhibition have been associated with increased AF propensity. Ozgen et al. first showed that fast pacing increases SK2 channel expression in rabbit atria (59). Subsequent studies have confirmed this finding in other species (rat, guinea pig, dog, goat, horse) and showed the protective effect of pharmacological *I*_SK_ inhibition (24, 33, 34, 36–40), which can also be mediated by secondary effects on *I*_Na_ properties (63, 64). On the other hand, Hsueh et al. (41) showed that *I*_SK_ block in dogs prolongs APD, facilitates the development of alternans, and is proarrhythmic. Reduction in SK2 and SK3 channel expression has been reported in a diabetic mouse model, resulting in APD prolongation and arrhythmias (25). Finally, increased AF susceptibility was found in both SK2 knockout mice (22) and mice overexpressing SK3 channels (35), the latter genotype being more prone to sudden cardiac death (65). The degree of AF-induced remodeling has also been shown to impact *I*_SK_ function, whereby studies in dogs and humans have shown that SK channel expression is reduced in cAF (23, 58, 66, 67), and *I*_SK_ block does not affect the APD. Other studies have instead reported increased *I*_SK_ in cAF (31, 49, 68). In our recent investigation, we did not find changes in SK channel expression at mRNA or protein level, but we demonstrated that *I*_SK_ is significantly increased in human cAF versus nSR, due to enhanced Ca^2+^-dependent SK channel gating and membrane trafficking and targeting (31).

Given the complex regulation of *I*_SK_ in human data, we did not include any AF-associated change in SK channel function during cAF simulations. Adopting the same nominal G_SK_ used for nSR, we determined that *I*_SK_ plays a primary role in the regulation of APD and ERP in the human atrial myocyte in cAF (Fig. 2). When repeating the same analysis accounting for a twofold increase in G_SK_ in cAF versus nSR (Supplemental Fig. S2), as suggested by our functional experiments (31), we found, as expected, an increased relative sensitivity to G_SK_ changes (see cAF coefficients in Supplemental Fig. S2 vs. Fig. 2). Interestingly, under this assumption, *I*_SK_ becomes the more influential K^+^ current for regulation of atrial myocyte repolarization and refractoriness (surpassing *I*_K1_). In our cAF simulations, we also assumed unchanged Ca^2+^-dependent activation of *I*_SK_ compared with nSR. Experiments in atrial cardiomyocytes from patients have shown an increased affinity for intracellular Ca^2+^ in cAF versus nSR (49), which could contribute to *I*_SK_ upregulation in the disease (31). Increasing Ca^2+^ affinity minimally impacts the results produced with the Grandi et al. model, especially in terms of regulation of APD, ERP, and CaT (Supplemental Fig. S3*A*). As discussed earlier, this is because SK channel function is controlled by the levels of Ca^2+^ in cleft and subsarcolemmal compartments that, during the AP, rise well above the *K*_d_ values used here. Our data revealed minor changes in the propensity for alternans and DADs, with the former being facilitated and the latter being counteracted by decreased *K*_d,SK_ (Supplemental Fig. S3, *B* and *C*). The limited impact of increasing Ca^2+^ affinity is confirmed with the 3-D model of Zhang et al. (Supplemental Fig. S4). Although BCL threshold for SCRs is not affected, decreasing *K*_d,SK_ lowers the BCL threshold for DAD in two (out of 10) atrial myocyte models at nominal G_SK_ (see Supplemental Fig. S4*A* vs. Fig. 5*A*). Taken together, these results suggest that enhanced G_SK_ and increased Ca^2+^ affinity can both contribute to SK channel gain of function and have a protective effect against cellular triggered activity. Based on these observations and preliminary data indicating increased *I*_SK_ in patients with paroxysmal AF (69), we speculate that *I*_SK_ upregulation can be an adaptive change to the increased propensity to DAD-mediated mediated cellular triggered activity that occurs during the early stage of AF when focal firing is thought to contribute to the periodic reinitiation of AF in the absence of clinically relevant ERP/APD abbreviation (70). As AF progresses toward a persistent (chronic) stage, SK channel gain of function turns into a maladaptive change that contributes to AF maintenance by causing reentry-promoting APD/ERP abbreviation (31, 71).

### Targeting SK Channels in Patients with cAF

Despite the contrasting results reported in the literature, inhibition of *I*_SK_ has emerged as a promising anti-AF strategy and is currently being pursued in a clinical trial (ID: NCT04571385) (72). Based on our findings, a successful completion of this trial would support the notion that the main mechanisms underlying AF are linked to reentry, rather than focal activity. However, the outcome of pharmacological treatment can be strongly affected by patients’ variability linked to stages of AF progression (discussed before), the presence of comorbidities, and sex differences. For example, AF often coexists with heart failure (HF), leading to a worse prognosis and more complicated patient management (73, 74). It has been shown that *I*_SK_ is upregulated in ventricular myocytes during HF (66, 67), where apamin can prolong the AP, but it is still not clear whether *I*_SK_ activation would either facilitate or counteract ventricular arrhythmias (75). Similar to our observations in atria, Terentyev et al. showed that upregulation of SK channels in HF attenuates DADs driven by spontaneous Ca^2+^ waves, thereby reducing triggered activity in ventricles (21). Bonilla et al. suggested that pharmacological *I*_SK_ inhibition in HF causes excessive AP prolongation, leading to repolarization instability and the development of EADs (66). On the other hand, the modeling study by Kennedy et al. showed that *I*_SK_ activation produces arrhythmogenic alternans and instabilities in single-cell and tissue simulations in ventricles (76). These observations suggest that SK channel inhibition in patients with AF and concomitant HF might induce additional side effects at the ventricular level.

It is well known that sex differences in cardiac electrophysiology exist and can affect pathophysiology and response to treatment (77). This also applies to AF, where several differences have been identified in prevalence, clinical presentation, associated comorbidities, and therapy outcomes in males versus females (78). Notably, the former are more likely to be diagnosed with AF at an earlier age, and the latter are more likely to have worse AF symptoms that typically last longer and are more severe (79). The mechanistic bases of these sex differences are still poorly understood, mostly because studies investigating sex-specific mechanisms of AF pathophysiology have so far been very limited (80). Experiments in rabbit ventricular myocytes revealed increased *I*_SK_ in female versus male animals (81). If these sex differences were confirmed in human atria, SK channel modulation might induce larger relative changes in female versus male myocytes, and if confirmed in human ventricles, SK inhibition could cause harmful AP/QT prolongation in females. Interestingly, no females (47 healthy male volunteers) were recruited in the successful *phase I* clinical trial NCT04571385 designed to test the safety and tolerability of AP30663 (72), an SK2 channel inhibitor that is showing antiarrhythmic efficacy (82).

### Limitations and Possible Extensions

In the present study, we have not explicitly considered the contribution of protein kinase A (PKA) and Ca^2+^/calmodulin-dependent protein kinase II (CaMKII) signaling. These pathways are important regulators of atrial myocyte function (83), and can synergistically promote atrial arrhythmogenesis (84). Since both PKA and CaMKII can affect SK channel function (31, 49), further studies are needed to explore the effects of *I*_SK_ modulation in the context of altered signaling in disease. We previously showed that the effects of *I*_SK_ inhibition on lengthening atrial myocyte APD and ERP are consistent between single cells and tissues (45). Moreover, our tissue simulations revealed that reduced *I*_K1_ promotes source-sink mismatch and triggered activity (84). Similarly, we expect that *I*_SK_ inhibition will not only increase single cardiomyocyte sensitivity to DADs (thus increasing the source), but will also depolarize the surrounding tissue and reduce the sink when cells are coupled, thus enhancing source-sink mismatch (84).

It has been shown that Ca^2+^ can block SK channels at IC_50_ of ∼20 µM (27), which can have profound effects in shaping *I*_SK_, as [Ca^2+^] can reach these levels in the submembrane domains, at least in ventricular cardiomyocytes (85, 86). When simulating Ca^2+^-dependent block, the predicted reduction in *I*_SK_ was small and occurred with the peak submembrane Ca^2+^, because [Ca^2+^] exceeds 5–10 µM only for a short interval at the beginning of the AP, with moderate consequences on atrial electrophysiology and arrhythmogenesis (Supplemental Fig. S5). Lastly, our atrial myocyte models were built assuming homogeneous distribution of SK channels through the plasmalemma (50). Experiments in rabbit ventricular myocytes suggested instead that SK channels are predominantly located near L-type Ca^2+^ and RyR2 channels (87). We found that increasing the density of SK channels in the junctional cleft compartment minimally affects our results (Supplemental Fig. S6). However, the inclusion of Ca^2+^-block has a more marked effect when SK channels colocalize with L-type Ca^2+^ and RyR2 channels (Supplemental Fig. S7), as a larger fraction of channels “senses” the Ca^2+^-dependent effect. Further studies assessing how this spatial proximity influences local and global Ca^2+^ signaling and *E*_m_ dynamics are clearly warranted.

### Conclusions and Future Directions

Our study shows a dual effect of SK channel modulation in human atrial cardiomyocytes in both nSR and cAF conditions. By counteracting reentry arrhythmias, *I*_SK_ inhibition may be suitable for the treatment of patients with cAF. This strategy, however, may cause an increase in vulnerability for cellular DADs, thus potentially promoting atrial ectopy. Nevertheless, if the increased ectopy caused by SK channel inhibition cannot induce reentry, which is opposed by the ERP-prolonging effect, the net outcome will be primarily antiarrhythmic. Overall, these observations are similar to those made for APD-prolonging *class III* agents. The use of these drugs that target the hERG K^+^ channel (responsible for the delayed rectifier K^+^ current *I*_Kr_) is constrained by the potential risk of inducing life-threatening ventricular arrhythmias through EADs (88). To better characterize the benefits and risks of *I*_SK_ modulation in patient subgroups, future research should investigate the contribution of SK channels to sex differences in atrial electrophysiology, remodeling, and arrhythmogenesis. In addition, efforts should be made to address the potential additional complications due to the concomitant occurrence of HF, whereby pharmacological SK channel inhibition might induce adverse side effects in ventricles, potentially in part mediated by altered mitochondrial function (89). Integrative computational modeling frameworks will allow comprehensive characterization of the effects of *I*_SK_ modulation [alone or in combination with other targets, as shown in (45, 90)] on male and female atrial and ventricular electrophysiology, thereby supporting the development of safe and effective therapeutical treatments against AF.

## DATA AVAILABILITY

All our source codes (and related documentation) used in this study and all simulated data presented here are freely available for download at the following links: elegrandi.wixsite.com/grandilab/downloads and github.com/drgrandilab.

## SUPPLEMENTAL DATA

10.6084/m9.figshare.23982561.v1 Supplemental Figs. S1, S2, S3, S4, S5, S6, and S7: https://doi.org/10.6084/m9.figshare.23982561.v1.

## GRANTS

This work was funded by National Institutes of Health Grants T32HL086350 (to N.T.H.), R01HL131517 (to E.G. and D.D.), and 1OT2OD026580-01, R01HL141214, and P01HL141084 (to E.G.); and R00HL138160 (to S.M.); American Heart Association Predoctoral Fellowship 20PRE35120465 (to X.Z.) and Postdoctoral Fellowship 20POST35120462 (to H.N.); German Research Foundation (DFG) Grant Do 769/4-1 (to D.D.); European Union (large-scale integrative project MAESTRIA) Grant 965286 (to D.D.); The Netherlands Organization for Scientific Research (NWO/ZonMW Vidi) Grant 09150171910029 (to J.H.); Norwegian Center for Research-Based Innovation Precision Health Center for Optimized Cardiac Care (ProCardio) Grant 309762 (to M.M.M.); and Burroughs Wellcome Fund-Doris Duke Charitable Foundation “COVID-19 Fund to Retain Clinical Scientists” award (to S.M.).

## DISCLOSURES

No conflicts of interest, financial or otherwise, are declared by the authors.

## AUTHOR CONTRIBUTIONS

E.G. and S.M. conceived and designed research; N.T.H. and X.Z. performed experiments; N.T.H. and X.Z. analyzed data; H.N., M.M.M., J.H., D.D., E.G., and S.M. interpreted results of experiments; N.T.H. and S.M. drafted manuscript; X.Z., H.N., M.M.M., J.H., D.D., and E.G. edited and revised manuscript; N.T.H., X.Z., H.N., M.M.M., J.H., D.D., E.G., and S.M. approved final version of manuscript.

## APPENDIX

The definitions of the parameters from the model of Grandi et al. are presented in Table A1.

**Table A1.** Definition of the Grandi et al. model parameters randomly perturbed to introduce variability in the population of models

Parameter	Definition
G_Na_	Maximal conductance of the fast Na^+^ current
G_NaL_	Maximal conductance of the late Na^+^ current
G_NaB_	Maximal conductance of the background Na^+^ current
G_to_	Maximal conductance of the transient outward K^+^ current
G_Kr_	Maximal conductance of the rapidly activating K^+^ current
G_Ks_	Maximal conductance of the slowly activating K^+^ current
G_Kur_	Maximal conductance of the ultrarapidly activating K^+^ current
G_Kp_	Maximal conductance of the plateau K^+^ current
G_K2P_	Maximal conductance of the two-pore domain K^+^ current
G_SK_	Maximal conductance of the small-conductance Ca^2+^-activated K^+^ current
G_K1_	Maximal conductance of the inward rectifier K^+^ current
G_K,Ach_	Maximal conductance of the acetylcholine-sensitive K^+^ current
G_CaL_	Maximal conductance of the L-type Ca^2+^ current
G_CaB_	Maximal conductance of the background Ca^2+^ current
G_ClCa_	Maximal conductance of the Ca^2+^-dependent Cl^−^ current
G_ClB_	Maximal conductance of the background Cl^−^ current
v_NKA_	Maximal transport rate of the Na^+^/K^+^ ATPase
v_NCX_	Maximal transport rate of the Na^+^/Ca^2+^ exchanger
v_PMCA_	Maximal transport rate of the plasma membrane Ca^2+^ ATPase
v_SERCA_	Maximal transport rate of the sarcoplasmic reticulum (SR) Ca^2+^ ATPase
v_RyR,rel_	Maximal transport rate of the SR Ca^2+^ release via ryanodine receptors (RyRs)
v_RyR,leak_	Maximal transport rate of the SR Ca^2+^ leak via RyRs
